# Special Issue ‘Minimally Invasive Urological Procedures and Related Technological Developments’

**DOI:** 10.3390/jcm10184225

**Published:** 2021-09-17

**Authors:** Bhaskar Somani

**Affiliations:** Department of Urology, University Hospital Southampton NHS Trust, Southampton SO16 6YD, UK; bhaskarsomani@yahoo.com

**Keywords:** kidney calculi, ureteroscopy, PCNL, renal tumour, AI, laser, TFL, urosepsis

The landscape of minimally invasive urological intervention is changing. A great number of new innovations and technological developments have happened over the last three decades, and this is reflected in the publication trends in Urology [1,2]. To address this topic, this Special Issue in the *Journal of Clinical Medicine (JCM)* is dedicated to collecting high-quality scientific contributions focusing mainly on technological developments in managing patients with small renal masses and kidney stone disease.

Two studies investigated the management of small renal masses [3,4]. The first study aimed to identify individual factors in ultrasound (US) that influence contrast-enhanced US (CEUS) image quality, to optimize further imaging workups of incidentally detected focal renal masses. Their findings showed that the focal image quality of CEUS examinations was impaired by a shrunken kidney, a large distance between the kidney and lesion from the body surface, and a smaller lesion size, while the exophytic growth of a focal renal lesion resulted in a better image quality. Awareness of these factors would allow for better patient selection and improve diagnostic confidence in CEUS. In the second study, the authors look at the role of single-site sutureless partial nephrectomy (PN) for small exophytic renal tumors [4]. Of the 52 patients who had laparoscopic PN (LPN), single-site sutureless LPN and traditional suture methods were performed in 33 and 19 patients, respectively. The warm ischemia time and the procedural time were significantly shorter in the sutureless group, showing that it is feasible with small exophytic renal cancer, with excellent cosmetic results and without compromising oncological results.

Several interesting findings were derived from the collective body of work on kidney stone disease (KSD). First, a comparison of holmium low 20W and high 60W Moses laser lithotripsy for ureteroscopy and laser fragmentation (URSL) for KSD was conducted [5]. The use of Moses high-power technology was significantly faster for lithotripsy and significantly reduced the operative time of the second procedure for patients to achieve a stone-free status, with the authors suggesting that a mid-power Moses technology laser was likely to set a new benchmark for treating complex stones, without needing a secondary procedure in most patients. With the advent of the Thulium fiber laser (TFL), the authors of another paper compared the risk of laser fiber fracture between the Ho:YAG laser and TFL with different laser fiber diameters, laser settings, and fibre-bending radii [6]. The authors bench-tested different lengths and radii of the 30WHo:YAG laser and a 50W Super Pulsed TFL, concluding that TFL appeared to be a safer laser with regard to the risk of fiber fracture when used in a deflected position.

Kidney stones are linked to metabolic syndrome (MetS) [7]. In one of the largest comparative cohort studies over a 19-year median follow-up, including 828 stone formers (SF) and 2484 age- and sex-matched non-SF, kidney stone formers were at an increased risk of developing MetS [8]. As stone disease is influenced by dehydration and warm weather [9], in the next paper, the authors looked at global variations in the mineral content of bottled still and sparkling water [10]. In this internationally collaborative study, they included 316 different still water brands and 224 different sparkling water brands. The authors conclude that as the mineral content of bottled drinking water varies enormously worldwide and as mineral intake through water might influence stone formation, bone health and CVD risk, urologists and nephrologists should counsel their patients on an individual level regarding water intake. The next paper on intervention for KSD looked at the incidence of acute kidney injury (AKI) post percutaneous nephrolithotomy (PNL) in a prospective observational study [11]. Of the 509 patients included, 47 (9.23%) developed postoperative AKI. A higher incidence of AKI was seen in older patients, with associated hypertension and diabetes mellitus, in those receiving ACE inhibitors with lower preoperative hemoglobin and higher serum uric acid, higher stone volume and density, multiple punctures and longer operative time. Patients with AKI also had an increased length of hospital stay, and 17% patients progressed to chronic kidney disease (CKD). The cut-off values for post-PNL AKI were patient age (39.5 years), serum uric acid (4.05 mg/dL) and stone volume (673.06 mm^3^). The paper highlights that the strong predictors of post-PNL AKI allow for early identification, proper counseling and postoperative planning and management, in an attempt to avoid further insult to the kidney.

Kidney drainage with percutaneous nephrostomy (PCN) is important in patients with advanced malignancies [12]. This was shown by the authors in their systematic review using 21 full-text articles including 1674 patients. PCN was performed for ureteric obstruction secondary to urological malignancies (37.8%), gynaecological malignancies (26.1%), colorectal and GI malignancies (12.9%), and other specified malignancies (12.2%). The average survival time post-PCN was 5.6 months and varied from 2 to 8.5 months across studies depending on the cancer type, stage and previous treatment. Their results showed that patients with advanced malignancies who needed PCN tended to have a survival rate under 12 months and spend a large proportion of this time in the hospital. They concluded that decisions about PCN must be balanced with survival and quality of life, which must be discussed with the patient. While extracorporeal shock wave lithotripsy (ESWL) treatment is used for KSD, in the next paper, the authors used extracorporeal shockwave therapy (ESWT) in patients with chronic prostatitis/chronic pelvic pain syndrome (CP/CPPS) [13]. From this perspective, a single-arm cohort study of a total of 215 patients, with an established diagnosis of CP/CPPS, underwent perineal ESWT once a week for six consecutive weeks with a protocol of 3000 pulses at an energy of 0.25 mJoule/mm^2^ and a frequency of 4 Hertz (Hz). Over 12 months, this study showed that ESWT was an outpatient, easy-to-perform, and minimally invasive procedure, alleviating pain and improving erectile function and quality of life in patients with refractory CP/CPPS.

Finally, the last two papers looked at the role of artificial intelligence (AI), which has quickly been growing in the field of urology [14,15,16]. The first paper looked at the role and impact of AI on urological diseases in a large comprehensive review of literature [15]. It covers the usage of AI in prostate cancer, urothelial cancer, renal cancer, reflux disease, reproductive urology, urolithiasis, paediatric urology and other endourological procedures. Furthermore, the role it plays in renal transplant, radiotherapy and robotic surgery is also covered in detail. The second paper on AI looked at a machine learning (ML) predictive model for post-ureteroscopy urosepsis in patients who needed intensive care unit (ICU) admission [16]. In this retrospective case–control study, the risk factors for urosepsis were predicted with reasonable accuracy by their innovative ML model. The authors conclude that focusing on these risk factors will allow clinicians to create predictive strategies to minimize post-operative morbidity.

Several interesting findings are derived from this collective body of work. While technological advances were addressed in combatting small renal masses and kidney stone disease, newer tools for diagnostic and surgical interventions were also covered. There are still many fundamental questions that need more evidence in order to be answered, relating to cost and quality of life management for these patients [17,18]. As the Guest Editor, I would like to give special thanks to the reviewers for their professional comments and to the *JCM* team for their robust support. Finally, I sincerely thank all the authors for their valuable contributions.
